# Lung transplantation in a woman with paraquat poisoning that led to pulmonary fibrosis—Widely reported by the media: A case report

**DOI:** 10.1097/MD.0000000000032263

**Published:** 2022-12-09

**Authors:** Yunxiao Wu, Ning Li, Suyan Li, Shumei Song

**Affiliations:** a Department of General Medical, Hebei General Hospital, Shijiazhuang, China; b Graduate School of Hebei Medical University, Shijiazhuang, China.

**Keywords:** a criminal case, lung transplantation, paraquat poisoning, the media

## Abstract

Paraquat is an extremely toxic herbicide with a high mortality rate on poisoning. It can damage vital organs, such as the lungs, liver, heart, and kidneys. In this study, we report a case of pulmonary fibrosis after paraquat poisoning in a patient who underwent a lung transplant procedure after preoperative administration of corticosteroids and immunosuppressive agents and continuous noninvasive ventilation support therapy.

**Patient concerns:**

An 18-year-old student was hospitalized owing to diarrhea, chest pain, and gradually evolving dyspnea.

**Diagnoses:**

Owing to the inability to estimate the intake concentration and dose, paraquat was only detected in the urine on the 13th day, resulting in rapid progression of the disease and severe pulmonary fibrosis.

**Interventions:**

Extensive media coverage has attracted the attention of all sectors of society. The patient received financial assistance; thus, she could receive a double-lung transplant with extracorporeal membrane oxygenation (ECMO) support on the 34th day after the poisoning.

**Outcomes:**

Postoperatively, the girl was actively rehabilitated, adhered to anti-rejection medication, followed up regularly, and had a good prognosis.

**Lessons:**

Lung transplantation is currently the most effective treatment for pulmonary fibrosis, and mass media campaigns can provide economic support, influence potential organ donation, and provide such patients more chances to survive.

## 1. Introduction

Paraquat poisoning can cause multiorgan dysfunction, with a mortality rate of 40% to 80%.^[1]^ This causes systemic reactions, such as pulmonary tissue edema and liver and kidney damage, owing to the production of oxygen free radicals^[2]^; however, there is no specific antidote. The most common pulmonary complications, emphysema and interstitial fibrosis,^[3]^ can lead to respiratory failure and even death.^[4]^ In this case, the girl was poisoned by her brother-in-law and the best treatment time was delayed, which led to irreversible lung damage and serious complications. Without the mediating role of new media, she had little chance of survival. New media plays a dominant role in patients’ reception of medical treatments.^[5,6]^

## 2. Case report

An 18-year-old female student was admitted to our hospital owing to diarrhea, chest pain, and gradually evolving dyspnea. The patient developed symptoms of diarrhea with intermittent nausea and vomiting after accidental ingestion of paraquat. Three days later, the patient developed a sore throat with chest tightness and shortness of breath. One week after poisoning, there were multiple ground glass shadows in both lungs with impaired liver and kidney function (glutathione transaminase 127 U/L, glutathione transaminase 143 U/L, creatinine 481.7 µmol/L and uric acid 588.4 µmol/L). For further treatment, the patient was admitted to our hospital with stable vital signs; body temperature: 36.5°C, blood pressure: 119/72 mm Hg, heart rate: 90 beats per minute, and respiratory rate: 19 breaths per minute. She had a decreased level of consciousness; chest auscultation revealed clear breath sounds in both lungs and no heart murmur. High-resolution chest computed tomography showed scattered patchy hyperdense shadows under the pleura of both lungs, and the patient received anti-infection and stomach-protection treatment. On day 9, the patient experienced chest tightness and shortness of breath on minimal exertion, productive coughs, difficulty swallowing, tongue pain, and inability to eat, regardless of loss of appetite or taste or any other cause. Ancillary tests suggested electrolyte disturbances. The patient had an altered mental status and received enteral nutritional support. The disease evolved, and severe pneumonia and respiratory failure developed, with oxygen saturation reducing to 67.5% and partial pressure of oxygen to 32.24 mm Hg. noninvasive ventilator-assisted breathing was provided, and the ventilator parameters were adjusted to 10 cm H_2_O for inspiratory positive pressure, 6 cm H_2_O for expiratory positive pressure, and 90% oxygen concentration. The true cause of the patient’s multiorgan damage was only determined on the 13th day when 70 ng/mL paraquat was found in the urine on toxicology testing. The patient developed diffuse interstitial lesions in the lungs, which were worse than those at the time of admission. The patient developed new complications with self-induced head pain and flushing. The patient’s respiratory condition gradually deteriorated while the patient was being treated with a combination of methylprednisolone and cyclophosphamide. Half a month later, her wheezing and breathlessness symptoms aggravated, lips were cyanotic, tongue was red, mouth opening was restricted; further, her breath sounds in both lungs were coarse and popping sounds could be heard on auscultation. Urine toxicology analysis was repeated; 0.55 µg/mL paraquat was detected. Paraquat and other poisons were no longer detected on urine toxicological analysis after day 19. On day 28, owing to the progression of pulmonary interstitial fibrosis, dyspnea worsened, oxygen saturation was <70%, and ventilator settings were gradually increased. On day 34, the patient was transferred to another hospital for bilateral lung transplantation under extracorporeal membrane oxygenation (ECMO) supportive care. After surgery, her vital signs stabilized; she was able to breathe spontaneously and weaned off ventilatory support and ECMO. The patient then underwent rehabilitation. More than a month after lung transplantation, the patient returned to our hospital for a follow-up examination, and no significant lung abnormalities were observed. The patient recovered well and has been able to live a normal life.

## 3. Discussion

Lung transplants are expensive and require long-term anti-rejection medication after surgery, and many patients give up treatment because they cannot afford this inflated cost. In addition, patients may not receive timely lung transplantation owing to the shortage of donor organs.^[7]^ The patient in this case showed symptoms of poisoning after being poisoned by her brother-in-law. The police arrested the suspect as soon as the cause was identified. This criminal case sparked a heated debate and was covered by social media. The patient’s treatment process received much attention because of media coverage, and the community provided assistance to the patient and eventually found a matching lung source within a short period. Through the green channel of organ transfer, a lung transplant was quickly performed and the patient gained her normal life back. Mass media can deliver information more effectively and efficiently and attract attention to events.^[8]^ The media coverage played a huge role in this story and provided the patient a chance to have access to medical treatment and financial relief. Therefore, in today’s online environment, maintaining effective communication with the media can help enable timely treatment of critically ill patients.^[9]^

Paraquat rapidly spreads to multiple organs, causing irreversible damage. Paraquat toxicity is most harmful to the lungs, mainly destroying the alveolar epithelium and causing acute pulmonary edema, which progresses to severe pulmonary fibrosis and respiratory failure within a few weeks and even leads to death.^[10]^ Anti-fibrotic drugs, including pirfenidone and nintedanib, have limited efficacy and can slow disease progression but cannot reverse the effects of the disease.^[11]^ Therefore, lung transplantation is the only effective means of treating pulmonary fibrosis and is the only measure that can improve the patient’s quality of life and reduce the mortality risk.^[12]^ Surgical options for lung transplantation include single-lung transplantation, double-lung transplantation, and lobar transplantation. Double-lung transplantation has a higher long-term survival rate. The patient in this study presented with interstitial lesions in the lungs, and double-lung transplantation was the best surgical procedure for treatment.^[13]^ Lung transplantation success depends on the number of toxic residues in the patient’s body. Complete removal of toxic substances can improve the success rate of transplantation, which helps avoid various morbidities such as infections and recurrence of interstitial fibrosis after transplantation.^[14]^ ECMO is a technique that supports cardiopulmonary function and can be used as an adjunct to lung transplantation to maintain oxygenation and provide supportive treatment for respiratory and circulatory failure.^[15]^

Gastric lavage, activated carbon adsorption, and blood purification can be used in the early stages to reduce paraquat concentrations in the body and prevent further absorption of the poison.^[16]^ Unlike previously reported cases of paraquat poisoning, the presence of paraquat in the urine was only detected in this case on the 13th day after poisoning; thus, the critical window for poison removal was lost. Some studies have shown the effectiveness of early combined glucocorticoid and immunosuppressive therapy, and patients were supported until day 34 in addition to the early application of methylprednisolone and cyclophosphamide.^[17]^ Additionally, clinically active treatment of complications such as impaired liver and kidney function, mediastinal emphysema, and respiratory failure can reduce damage to other organs.^[18]^ Although the above treatment can alleviate the disease, it cannot prevent progression of pulmonary fibrosis. Therefore, lung transplantation is the only treatment that can improve the patient’s condition. Currently, there is a shortage of lung sources and a high degree of requirement for donor–recipient matching. Many patients have given up treatment because they cannot wait for a lung source. Fortunately in this case, with extensive media coverage, the patient was quickly matched with a suitable lung source and transferred to a higher-level hospital for treatment. Professor Jingyv Chen, a lung transplant specialist, reviewed the online report and arrived at the PLA General Hospital in Beijing overnight to perform the lung transplant.

## 4. Conclusion

This report describes the case of an 18-year-old girl who was poisoned by her brother-in-law, owing to which she experienced pulmonary fibrosis and underwent lung transplantation. Given extensive media coverage, the patient was quickly matched to a lung source and her life was saved. In clinical practice, the possibility of poisoning needs to be considered in cases of interstitial pulmonary fibrosis of unknown origin. Early detection and removal of toxins cannot be missed. Finally, media coverage can provide patients with safe and rapid access to treatment.

## Acknowledgments

The authors thank Wolters Kluwer Academic Services (www.aimieditor.com) for English language editing and review services.

## Author contributions

**Investigation:** Shumei Song.

**Resources:** Suyan Li, Ning Li.

**Writing – original draft:** Yunxiao Wu.

**Writing – review & editing:** Ning Li, Shumei Song, Suyan Li.
